# Kentucky and Indiana State Dental Associations

**Published:** 1873-07

**Authors:** 


### AMERICAN JOURNAL

OF

### DENTAL SCIENCE.

Vol. VII. THIRD SERIES--JULY, 1873. No. 3.

### ARTICLE I.

### *Kentucky and Indiana State Dental Associations.*

The Association for the "Protection of the Rights of Dentists, of Kentucky," against illegal patents, met Wednesday morning, June 4, at 9 o'clock, with Dr. Goddard presiding. The meeting was a large and enthusiastic one, and a number of new members were added to the roll.

After listening to some interesting remarks by Dr. Keely, of Ohio, which were followed with some discussions of the subject by various members, transacting some unimportant business, the Association adjourned, subject to the call of the President, having been in session about forty minutes.

#### Morning Session.

After waiting some time for the arrival of the Indiana Association, the joint meetings of the State Dental Association of Kentucky and Indiana was called to order at half-past ten o'clock.

After prayer by Rev. J. H. Heywood, the president of the Kentucky Dental Association welcomed the Indiana Association in the following words:

*Mr. President, and Gentlemen of the Indiana State Dental Association.*

It becomes my official duty to extend to you, in behalf of the Kentucky State Dental Association, a welcome to our State, our city and our fraternal hospitalities; and I fulfil the duty with all my heart; for in this case, official duty becomes the highest personal privilege. Yes—I regard it as my personal good that I am the organ of our Association in offices of friendship and courtesy which enlists the warmest sympathies of which I am capable.

Accept then, Mr. President, for yourself and your associates, the welcome which I extend to you; and believe me, the cordial words of greeting which I utter are truthful exponents of our feelings. Our hearts are on our lips, and in the grasp of our hands. Welcome among us—thrice welcome! Welcome to our homes, our fellowship, our hearts! We thank you for the opportunity you have given us, of evincing our fraternal regards; and we trust that when you leave us to return to your homes, it will be with the bands of amity and friendship between our two societies more strongly cemented than before, and with personal ties renewed or freshly created which shall not be sundered this side the grave!

By the programme of arrangements for our order of business, we are to join together as one body for discussions and interchanges of thought and experience, and to that interesting feature of the occasion let me devote a few passing words.

I am irresistibly led to notice the delightful contrast which the circumstances of this occasion present to the spirit which in bygone years marked the prevailing attitude of professional brethren, each to each. For it is but a few years since each one of us considered it a duty to himself to look with suspicion on every professional brother, to conceal every new thought, every new discovery, closely within his own breast, or his own laboratory; yes, even, to lock his door against the entrance of a brother dentist, who might, perhaps, if admitted to confidence and intercourse, pillage some of the methods on which his host prided himself, and use them in his own operations.

There was no fraternity of feeling, no reciprocity of good offices, no free interchange of suggestion and experience. But we have come to feel, thank God, that all this was contemptible by-play, unworthy of men of thorough scientific knowledge and progress. We have come to believe that when any man throws his contributions of thought, experience and skill into the common stock, he grandly benefits himself as well as the world at large. This magnanimity and philanthropy return home to bless him more abundantly. Fraternity is now the word!

Knowledge loves to have the votaries who sit reverently at her feet clasp each other's hands in a close embrace as they do so, for her sake, for the world's sake, and for their own sakes! By as much as *we* claim to be lovers of truth and lovers of science, above the petty arts of the selfish and narrow pretender, we glory in the intercommunication of of knowledge and good offices. Now all are brothers; we affiliate in associations; we meet to impart all we know to each other, and to put each other, if possible, on a higher plane of power and opportunity. We do not fear our instruments, our materials, or our knowledge will be stolen from us. On the contrary, our offices and our laboratories, with all they contain, are open to the examination of every worthy brother. Yes, and our hearts and purses are open too.

It is interesting to note that this is only the second instance of the union of two State societies in joint sessions for mutual discussion and improvement; that of the Illinois and Iowa associations, who lately met together, having been the first. This is the second; may it not be the last! But may the exercises of this occasion so bind us together that we shall be eager for a repetition of the genial and profitable fraternity, and relations be this day inaugurated which shall be renewed with ever-increasing lustre and profit, so that in the far future our successors shall rise up and call us blessed! Once more, Mr. President, I tender you a most cordial welcome.

In response, the president of the Indiana Dental Association, Dr. Wells, returned thanks in the name of the members of the association which he represented to the Kentucky Association for the kindness and fraternal feeling which had been shown toward them, which they should not fail to reciprocate when the occasion offered. They had come to seek knowledge and light with their Kentucky brethren. Although separated by geographical lines, they were united in bonds of fraternity and affection, and hoped they would continue ever to be so.

Dr. Priest read the minutes of the previous meeting, which were approved, when the following subject, the first on the programme, was announced as in order :

"Contour filling—what are its advantages, what its permanency, and what the essential points to be observed ?"

Dr. P. G. C. Hunt, of Indianapolis, who was selected by the executive committee to open the discussions on this point, arose and stated that he had with him a few casts of cases which had come under his treatment. He preferred to change the subject a little, and would take his starting point at the last clause, namely ; "What are the essential points to be observed ?" The main subject under consideration was contour filling, or restoring a tooth to shape. He treated at some length of his methods of proceeding, saying that when a tooth is broken down, in whole or in part, the dentist must draw largely on his ingenuity. He presented casts of cases in which the teeth had been largely broken down, the teeth as restored often appearing to be principally constructed of gold. It is necessary to first remove all deceased parts, or all decayed bone, so as to leave a healthful border for the filling. The next step is to so secure the gold that there is no danger of its becoming loose. Screws he regarded as very valuable in some cases to secure contour filling, but he does not regard them as absolutely necessary in any case. He believed in letting the lateral enamel stand for lateral support for the filling. In many cases he fastened the filling by inserting gold wire in the root, an arrangement which he regards as more cleanly and more durable than the pivot tooth.

The advantages of contour filling are that it presents a smooth surface to the tongue, a better surface for mastication and for articulation.

In regard to permanency he had not much experience. He had been using the contour filling for three or four years, and found that it would wear, but not so rapidly as the two surfaces of bone would.

Dr. Hunt continued his subject at some length describing processes and material. The discussion which followed took a wide range; over all the ground covered by the qnestion as stated.

On motion the Association agreed to meet at 9 o'clock, and adjourn at half-past 12 o'clock in the morning, and to meet at 3 o'clock and adjourn at half-past 6 in the evening.

After further discussions of the question presented for the morning session, the Associations adjourned at half-past twelve.

### Afternoon Session.

The Associations were called to order by the President of the Kentucky Association, Dr. W. H. Goddard, at twenty minutes after two o'clock.

The consideration of the second subject was passed over, as no member had any remarks to offer.

The third subject was taken into consideration. This subject was stated as follows:

The importance of preserving the vitality of the teeth, and what are the best means for securing it.

Dr. Taft, of Cincinnati, read the following paper on the subject of diagnosis in practice, by Dr. D. C. Hauxhurst:

### DIAGNOSIS.

I have hinted at some of the difficulties to be met with in tracing symptoms to their causes in a former number of this journal. Before developing the differential diagnosis of special affections, it seems proper to offer a word on the sympathetic relations of the different parts of the organism.

The intricacy and obscurity of the numerous cross-lines of sympathy which bind together the parts and functions of the body, declaring themselves with especial emphasis during disease, often prove very embarrassing to the practitioner, and sometimes constitute his principal hindrance to a correct diagnosis. The files of our dental journals contain numerous illustrative cases.

The direct response which the teeth makes to changes in the blood and to states of nutrition, and the reverse action by which causes at work in the teeth, produce serious and startling, often intensely damaging effects upon the blood and other parts, are so obvious as scarcely to require mention.

If causes are set in motion in the nervous system sympathetic action spreads, and not unfrequently fall into channels of communication leading to the dental organs, from which reflective waves of influence beat back upon the general system.

The mixed character of these effects, and the embarassment which the practitioner who does not understand the sympathetic relations of the parts of the body, must meet with in attempting a diagnosis, serve to impress their importance.

In diagnosing a case it should be remembered that the animal body is an organic unit, whose parts are mutually dependent, and that by consequence disease in any one organ is liable to introduce morbid action into any other, or even many others. As an insignificant atom of dust may disturb the motions of every wheel in a watch, so a morbific agent introduced at any point in the organism may seriously disturb or entirely arrest action in the entire machinery of the animal body.

It should now be called to mind that diseased action is transmitted through certain channels more readily than others; that it is obedient to laws of sympathy and susceptibility on the part of organs to which it is transmitted; and that slight and transient causes may divert it to unaccustomed channels, and to unusual tissues and organs.

In diagnosing cases it is necessary to know something of those relations and their causes; otherwise I know not how we shall be able to trace the usually obvious symptoms to their often entirely obscure pathological causes, and form in the mind an accurate picture of the extent, location and character of the disease.

In some instances the symptom brings with it the evidence of its distant origin, and the path backward may be easily traced. But more often the dependence of the part which makes the outcry and contains the only visible sign of disease, upon some other and undeclared terrritory of morbid action, is quite hidden. Neither our own nor the patient's senses can point it out to us. It is only to be approached by a consecutive chain of reasoning, on the basis of the laws of sympathy and relation between the parts of the animal body.

And no matter how well we are armed with a knowledge of these laws, we will often meet with the most embarrasing difficulties. The clue which we have been following by a careful logic, backwards to the source of trouble, sometimes ends suddenly in a tract, entirely barren of evidence. It is obvious that he who best understands physiological and pathological phenomena, and can most skilfully trace their relations, will be best prepared to make a difficult diagnosis.

It is worthy of remark that while much has been written in a careless way about the sympathies of organs and parts with each other, but little had been done toward tracing the laws of this sympathy; until this is done our efforts at diagnosis must often be blind and misdirected.

We have illustrative cases on record. Our case-book and journals are full of cases indicating special extraordinary sympathies of the teeth and associate parts with distant organs, functions and pathological conditions.

In this way the eye, the ear, the scalp, the neck, shoulders, the stomach, liver, spinal cord, the limbs along with the functions of fœcal excretion, menstruation, gestation and lactation have been pointed out as sympathizing at times with conditions of the teeth, while epilepsy, diseases of the antrum, hysteria, trismus, uterine affections and several important nervous diseases have been shown to possess unmistakable connections with their pathological states.

But for the most part the cases on which these deductions rest have not been carefully studied and recorded. Their history has been imperfectly given; the general state of health in the patients has received little attention; diathesis has been largely ignored; and many facts which it would be exceedingly desirable to know have been omitted.

I have in my mind some admirable exceptions, but they are rare in the extreme. It is evident that before we can have any very complete development of those special forms of pathological sympathy, which must in part constitute the basis of diagnosis in our profession, we must learn how to observe and record cases.

Those already recorded are highly valuable, but might have been much more so. They have pointed out to us special sympathies, and in many instances have furnished hints toward general laws of sympathetic actions. What important conclusions may be drawn from a careful classification and comparison of these materials, remain to be seen. It is evident that the attainments of general principles must be facilitated by whatever of accurate observations the future may record for us.

In this article I have only aimed to throw out a few hints to put the young practitioner upon the right method of diagnosis. Any accurate statement of the laws of that immensely complicated and intricate weft-work which makes up the sympathetic relationship of tissues, organs and functions, belongs of course to the differential diagnosis of special diseases, and would be best made in connection with the phenomena of each.

This subject he considered a very important one, relating to daily practice of every dentist. Teeth are affected by causes remote from the mouth. These cases are difficult to treat without knowing these remote causes. Disorder of other organs will extend their influence to the pulps of the teeth whether exposed or not. In some cases of uterine irritation acting upon the teeth, it is difficult to trace the action or to understand the cause. This action Dr. Taft illustrated with various cases which he has treated. Other nervous affections will extend their influence to the teeth and the parts around them. Dentists should understand well the pathology of the system. It should be a subject of study with them; otherwise they are laboring in the dark. Considering the ignorance of some dentists on this point it is no wonder their best operations often failed.

Dr. Taft continued on this subject for some time, endeavoring to show the necessity to the dentist of thorough knowledge of the human system.

Considerable discussion of the subject followed, and many interesting instances were adduced of cases bearing on this point.

The third question was taken up for discussion:

Importance of conserving the vitality of the teeth, and what are the best means of securing it?

Dr. Taft led the discussion on this point, a discussion which was participated in by most of the members.

The chair announced the following operatives for the clinics of the following morning: Drs. Keely, S. Brown and Merritt Wells, of Indiana.

Also, that at half-past 4 o'clock the next (third) day Dr. Seymour would fill a tooth at his office, on Chesnut street, between Second and Third, by request.

Dr. W. F. Morrill then read the following paper:

### THE DENTIST OF THE FUTURE.

American dentistry, from the earliest annals down, must be conceded to be of significance, interest and profit. Casting our eyes over that period when dentistry first began its existence, we recall the giants—not pigmies—who lived in those days. Among these were Gardette, Greenwood, Harris, Palmby, Townsend, Badger, Maynard, and others, who became fixed stars in this firmament of pioneer dentistry, and whose effulgence still glows with a steady, ineffaceable radiance. We venerate their names and their deeds, for they were glorious. Conspicuous as these bright examples are in our history, conspicuous as many illustrious which still live to adorn our profession to-day, we cannot resist the temptation of throwing forward the horoscope, and of snatching a gleam of what shall be *the dentist of the future.*

Comparatively unknown to the pioneers of dental practice, were the researches of science and the ingenuity of art now made subservient to our purposes. The precious germs of knowledge, once so scarce, have multiplied and been spread broadcast in our day, so that each representative of our vocation may increase his store of information without stint or hindrance. Notwithstanding these sunny avenues of our intellectual life, sweet and attractive as the palm colonnades of a tropical clime, where the fountains of truth scatter their germs, the number of dental practitioners who wander in these grateful retreats, and pause to sip the waters of science are, alas, too few. We could wish that responsibilities of the future, which is to be a period not of apathy, but of industry and science, could be more evenly distributed.

Defective education, formidable and wide-spread, besets our progress. But the echoes of reform, reform, reverberate from the shores of the Atlantic to the slopes of the Sierras along the Pacific.

The exigencies of the times, the requirements of humanity, alike demand that he who would reach the *ultima thule*

of our profession, must possess high attainments. Broader and more thorough culture is demanded. A preliminary education, high and liberal, is an indispensable requisite, as much so as in other professions. The curricula of our dental colleges are tending toward improvement and excellence. The training and discipline necessary for a candidate to receive the honors of these valued institutions, and to fit him to enter upon an honorable career of dental practice, must be painstaking and scrupulously exact.

It will be no disparagement to our illustrious prototypes who have adorned our profession, nor to those who still grace it with becoming honors, to say that the dental practitioner of the future must possess greater qualifications than his predecessors. Much broader and more catholic principles, and more unselfish tenets prevail to-day among dentists than at any previous time in our history.

Prejudices and petty bickerings among the cultivated as well as among the unlearned of our calling are slowly disappearing; while men, freer and more enlightened in their moral nature, are encouraging social and friendly intercourse. The free offerings of our collective counsels and experience, which these associations themselves impart, like the irrepressible light and air of heaven, infuse fresh air and energy into the monotonous existence of every practitioner. These organizations, binding together with adamantine chains professional and fraternal sympathy, that are the instrnmentalities that are destined to encompass the whole land with their recuperative energy. These are the assurances written upon the lintels of our profession. We are not, then, left in doubt as to what shall be the qualifications of him who is to be *the dentist of the future.*

The sphere of scientific research expands daily. An acquaintance with all the minute and exact discoveries of medical science should be the laudable ambition of every practitioner of dentistry who expects large results. There is no one with a brilliant record here to-day who does not possess in a large degree this culture. These observations, made richer and more valuable by experience as time rolls on, will become the inheritance of our successors. More natural and rational modes of treatment will then be understood and practiced. The diagnosis will be accurate, penetrative, discerning. The utility and value of the teeth in their relations to human economy, to say nothing of their beauty, will be better appreciated, even among dentists. Conservative methods for their preservation will be more persistent, and rewarded with higher triumphs. Three-fourths of the cases we see nowadays, deformed with hideous artificial teeth, under this new guidance and superior wisdom, might be wearing their own natural teeth. Labor saving machines and mechanical appliances, far beyond anything we now know of, will continue to be brought to higher perfection and greater utility. And these facilities, turned into the celerity and ease of operations on the teeth, will increase the character of work performed. Our text books, embracing the results of scientific discoveries, will furnish valuable assistance to the student of the future, containing as they will, digested principles of dental science. In this list of subjects, treated of in standard works, will be: dental physiology, dental pathology, dental microscopy, and anatomy, dental therapeutics, dental chemistry, dental surgery, and dental operations by a score of authors. It must be confessed with no little mortification that few works of this kind at present adorn the shelves of our libraries. All the advantages to be derived from this service, apparently so indispensable to our present needs, will be reserved for our more fortunate successors. The current periodical literature of our profession, now so often fragmentary, loose, ephemeral, will have a more solid and enduring character. Those who shall hereafter have charge of this department, will be men who can devote all their time and attention to this one pursuit.

In taking part in these triumphs of our profession, the student will have claim to rank, fellowship and recognition among the learned. In the domain of art, which is an indispensable adjunct to the specialty of dentistry, the results reaching higher ideals in expression, and continuing the assimilative and fusing processes of the imagination—methods far beyond any now known—will be the tempting of the future votaries of our calling. It is almost useless to predict what is fast becoming an accomplished fact: that of making the manufacture of artificial dentures a separate department. Even now there are numerous dental practitioners who devote themselves entirely to operative dentistry, lopping off the mechanical branch so far as dentures are concerned. This indicates the exercise and concentration of all the powers and faculties in one given direction, thus increasing the efficiency of this department.

With all the civilizing forces and world-moving tendencies urging us to duty and to truth, we can neither remain idle nor listless spectators, but earnest co-workers, to carry forward the great interests of dentistry which are to have such an important bearing in the future.

The great want of the present, as auxiliary to this means of preparation, is the tone of public sentiment. If we had some way not degrading, by which we could communicate hygienic laws to the masses; make them acquainted with the nature and value of dental operations; we might lay aside some of the dignity of our calling, and invade even the confines of our ethics to have this information given. Under proper guidance and judgment the channels of the press might be employed. The clergyman does not rely upon supernatural powers alone to keep the truths of revelation alive, but arouses the people to a due respect for the lessons they teach. The mission of a dentist may not have quite this eternal importance, but, during our sojourn on earth, it is some comfort and happiness to know that adequate skill to preserve the human teeth through a natural lifetime is fast attaining toward these perfections. Time in our offices, to impart instruction to patients with regard to hygienic laws specially adapted to the teeth, is too limited. Every dentist knows what comfort and ease he experiences when he approaches a patient accustomed to dental operations; but if he has ignorance and skepticism to baffle with, how laborious he himself becomes, and how unsatisfactory are his services. True, each year carries us forward to a better condition of public appreciation of the dentist's services, yet these requirements are not all filled. The practice of our calling should have less discomforts than it has to-day. Punctuality and regularity in the attendance of patients on their engagements is not near so prompt as it should be. In the success of the practitioner of the future, it will be more esteemed a duty, and not so much dread to pay visits to his office. It will not then be for surgical or mechanical operations that these are paid, but for the purpose of gaining such advice as shall place the patient beyond the need of operations from dentists' hands; such are found in hygienic laws. Public sentiment, after these advances of our profession have been made, will be more elevated toward us, and not hesitate, as is now often the case, between the meritorious and pretender.

Empiricism will have nothing to feed its dupes. Legislation for protecting the people against quackery in our profession will be needless. The scope of his opinions will not only embrace the present wants, but extend from etiology to an assured prognosis. In a word, the dentist of the future will be the acclaimed counselor and custodian of the teeth.

These prospects, rather Utopian, perhaps, I have abiding faith to believe, are but imperfectly hinted. Nevertheless, as a co-worker upon the living tissues of that mystic life to which we are mainly dedicated, I bid you, not in the burning words of Napoleonic grandeur, "that forty centuries look down upon you;" but from the heights of the past and present ages, "countless worthies of our godlike profession point and beckon to a goal more elevated than attracts legislators and conquerors, Solons and Cæsars."

It was moved that a Publication Committee, consisting of three members—two from Kentucky and one from Indiana—be appointed. The motion was carried, and the President announced that he would name the committee the next day.

The joint session then rose, and the Kentucky Association went into an election of officers, the following ticket being elected: Drs. Priest, President; Rogers, Vice President; Doyle, Secretary; Canine, Treasurer. Board of Censors, Dr. Goddard, vice Dr. Redman, term expired.

Executive Committee—Drs. Priest, Canine and Dunn, re-elected.

Adjourned.

